# Effects of ketoisocaproic acid and inflammation on glucose transport in muscle cells

**DOI:** 10.14814/phy2.14673

**Published:** 2021-01-05

**Authors:** Gagandeep Mann, Olasunkanmi A. J. Adegoke

**Affiliations:** ^1^ Kinesiology and Health Science and Muscle Health Research Centre York University Toronto ON Canada

**Keywords:** BCAAs, BCAT2, BCKD, insulin resistance, insulin sensitivity, skeletal muscle

## Abstract

Branched‐chain amino acids (BCAAs) are regulators of protein metabolism. However, elevated levels of BCAAs and their metabolites are linked to insulin resistance. We previously demonstrated that the leucine metabolite, α‐ketoisocaproate (KIC), inhibited insulin‐stimulated glucose transport in myotubes. Like KIC, inflammatory factors are implicated in the development of insulin resistance. Here, we analyzed the effect of KIC and inflammatory factors (homocysteine [50 μM], TNF‐α [10 ng/ml], and interleukin 6 (IL‐6) [10 ng/ml]) on myotubes. Although KIC suppressed insulin‐stimulated glucose transport, addition of the inflammatory factors did not worsen this effect. Depletion of branched‐chain aminotransferase 2, the enzyme that catalyzes the conversion of leucine into KIC, abrogated the effect of KIC and the inflammatory factors. The effect of insulin on AKT^S473^ and S6K1^T389^ phosphorylation was not modified by treatments. There were no treatment effects on glycogen synthase phosphorylation. Depletion of E1α subunit of branched‐chain α‐keto acid dehydrogenase, the enzyme that catalyzes the oxidative decarboxylation of KIC, suppressed insulin‐stimulated glucose transport, especially in cells incubated in KIC. Thus, defects in BCAA catabolism are contributory to insulin resistance of glucose transport in myotubes, especially in the presence of KIC. Interventions that increase BCAA catabolism may promote muscle glucose utilization and improve insulin resistance and its sequelae.

## Graphical abstract

Whether amino acid metabolites cause or worsen insulin resistance is not clear. While KIC (a metabolite of leucine) caused insulin resistance, its effect was not worsened by inflammatory factors. Depletion of BCKD (which decarboxylates KIC) worsened insulin resistance, especially, when KIC was present.

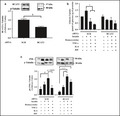

## 1 INTRODUCTION

Type 2 diabetes mellitus (T2DM) is a chronic metabolic disease that is now regarded as an epidemic in some countries (Lovic et al., 2019; Olokoba et al., 2012; Unnikrishnan et al., 2017). Insulin resistance is the strongest predictor of developing T2DM (Bunt et al., 2007), thus, targeting insulin resistance can help prevent the development of T2DM (Fujimoto, 2000).

Dietary protein and, in particular, branched‐chain amino acids (BCAAs; leucine, valine, isoleucine) stimulate muscle protein synthesis and regulate body weight and glucose homeostasis (Lynch & Adams, 2014). However, increased circulating levels of BCAAs are present in insulin‐resistant states like obesity and T2DM, and BCAA levels are predictive of future insulin resistance (Andersson‐Hall et al., 2018; Lackey et al., 2013; Lian et al., 2015; Mccormack et al., 2013; Newgard et al., 2009; Wang et al., 2011). Furthermore, circulating levels of branched‐chain α‐ketoacids (BCKAs, metabolites of BCAAs) are elevated in insulin‐resistant individuals (Adams, 2011; Giesbertz et al., 2015; Mccormack et al., 2013; Newgard et al., 2009). These observations raised the question of whether increased levels of BCAAs and BCKAs cause insulin resistance, or ARE symptoms of insulin resistance. α‐ketoisocaproic acid (KIC, the ketoacid of leucine) suppresses insulin‐stimulated glucose transport in L6 myotubes (Moghei et al., 2016). This effect is attenuated when branched‐chain aminotransferase 2 (BCAT2), the enzyme that catalyzes THE reversible conversion of leucine into KIC, is depleted. In addition, skeletal muscle mRNA levels of BCAT2 and of the E1β subunit of branched‐chain α‐ketoacid dehydrogenase (BCKD), the enzyme complex that catalyzes the irreversible oxidative decarboxylation of branched‐chain α‐ketoacids, are reduced in skeletal muscle of T2DM patients (Hernández‐Alvarez et al., 2017). In liver of type 2 diabetic rats (Kuzuya et al., 2008) and adipose tissue of db/db mice (Lackey et al., 2013), protein levels of BCKD subunits are decreased. In addition, BCKD activity is downregulated in type 2 diabetic mice liver, adipose tissue, and skeletal muscle (Lian et al., 2015). These data suggest a role for muscle, liver, and adipose tissue catabolism of BCAA in the development of insulin resistance. It remains to be seen if reduced BCKD expression is causative.

Pro‐inflammatory factors too are implicated in the development of insulin resistance (Shoelson et al., 2006). Tumor necrosis factor‐α (TNF‐α) (Feinstein et al., 1993; Hotamisligil et al., 1993), IL‐6 (Kim et al., 2009), and homocysteine (Li et al., 2008; Wang et al., 2000, 2002) reduce insulin signaling.

Skeletal muscle is the main site of insulin regulation of glucose metabolism (Thiebaud et al., 1982). We previously demonstrated a negative effect of KIC on insulin‐stimulated glucose transport in myotubes (Moghei et al., 2016). Here, our objective was to examine whether inflammatory factors would modulate the effect of KIC and if such modulation would depend on cellular level of BCAT2. We also asked whether manipulation of BCKD level would affect insulin‐stimulated glucose uptake. We showed that whereas KIC suppressed insulin‐stimulated glucose transport in myotubes, this was not modified by co‐incubation with pro‐inflammatory factors. The effect of KIC or pro‐inflammatory factors was attenuated when BCAT2 was depleted. We also demonstrated a significant reduction in insulin‐stimulated glucose transport in cells depleted of BCKD, especially in myotubes incubated with KIC, consistent with the assertion that defects in muscle BCAA catabolism is causative for insulin resistance.

## 2 MATERIALS AND METHODS

### 2.1 Reagents

Alpha modification of Eagle's medium (AMEM), phosphate‐buffered saline (PBS), trypsin, and antibiotic–antimycotic preparations were purchased from Wisent (St Bruno, Quebec, Canada). Fetal bovine serum (FBS), horse serum (HS), Lipofectamine RNAiMAX, and Opti‐MEM 1X Reduced Serum Medium were purchased from Thermo Fisher Canada (Burlington, Ontario Canada). Amino acid‐free RPMI 1640 medium was purchased from US Biologicals (Salem MA). Sodium 4‐methyl‐2‐oxovalerate (sodium salt of KIC), 2‐deoxyglucose, protease and phosphate inhibitor cocktails, anti‐BCAT2 and anti‐gamma tubulin antibodies, siRNA oligonucleotides, amino acid standard, o‐Phthalaldehyde, interleukin‐6, and homocysteine were purchased from Sigma Aldrich (Oakville, Ontario, Canada). Phospho (ph) ribosomal protein S6 kinase 1 (S6K1) (T389), ph‐ribosomal protein S6 (S6) (S235/236), ph‐Akt (S473), ph‐SAPK/JNK (T183/Y185), ph‐glycogen synthase (S641), BCKDH‐E1α, horseradish peroxidase (HRP)‐conjugated anti‐rabbit and anti‐mouse secondary antibodies were purchased from Cell Signaling Technology (Danvers, MA). [^3^H]‐2‐deoxyglucose was purchased from Perkin Elmer (Markham, Ontario, Canada) while chemiluminescence substrate was from Millipore (Etobicoke, Ontario, Canada). TNF‐α was purchased from Shenandoah Biotechnology (Warwick, PA).

### 2.2 Cell Culture

L6 rat skeletal muscle myoblasts were purchased from American Type Culture Collection. Cells were cultured in 10‐cm plates with growth medium (GM: AMEM supplemented with 10% FBS and 1% antibiotic‐antimycotic preparations) as described before (Moghei et al., 2016). Cells were seeded (2 × 10^5^ cells/well) in six‐well plates for western blot experiments or (10^5^ cells/well) in 12‐well plates for glucose transport experiments. They were allowed to proliferate for 48 hr or until they became 90–100% confluent. They were then shifted into differentiation medium (DM: AMEM, 2% HS, 1% antibiotic–antimycotic preparations) and replenished with fresh DM every 48 hr. Myotubes were used on d 5 or d 6 of differentiation.

### 2.3 siRNA gene silencing

On d 3 of differentiation, cells were transfected with 50 nM of BCAT2 siRNA oligonucleotides (sense 5′‐CUAUGUGCGGCCGGUGCUU, anti‐sense 5′‐AAGCACCGGCCGCACAUAG), or 50 nM of scrambled siRNA oligonucleotides using Lipofectamine RNAiMAX reagent according to the manufacturer's instructions. Twenty‐four h after transfection, DM that contained pro‐inflammatory factors (homocysteine [50 μM], TNF‐α [10 ng/ml] and IL‐6 [10 ng/ml]) was added to each well (1 ml per well for six well plates; 0.5 ml per well for two well plates) for 48 hr to produce a chronic inflammatory response (Aguirre et al., 2000). We used these reagents at indicated concentrations because homocysteine promotes the activation of inflammatory pathways both in vitro and in vivo (Wang et al., 2000, 2002) and hyperhomocysteinemia ranges between ~15 and 50 μM in humans (Lipton et al., 1997). TNF‐α activates inflammatory responses at 0–10 ng/ml concentration range (Bhatnagar et al., 2010; Ciaraldi et al., 1998), while IL‐6 induces the production of pro‐inflammatory cytokines at 10 ng/ml (Romano et al., 1997). Myotubes were then starved of amino acids for 3 hr and then supplemented with or without KIC for 30 min. Incubation then continued with or without insulin for another 20 min. Cells were afterward used to assay glucose transport or were harvested for western blotting. Where indicated, cells were transfected with siRNA oligonucleotides designed against E1α subunit of BCKD (sense 5′‐CAGAUCGUGAUCUGUUACU, anti‐sense 5′‐AGUAACAGAUCACGAUCUG) as described above. Two days post transfection, and after 3 hr starvation in amino acid‐free medium, myotubes were supplemented with or without KIC for 30 min. They were then treated with insulin and afterward used to assay glucose transport or were harvested and processed for western blotting.

### 2.4 Glucose transport

Following treatments, myotubes cultured in 12‐well plates were washed twice with HEPES [4‐(2‐hydroxy‐ethyl)piperazine‐1‐ethanesulfonic acid]‐buffered saline]. They were then incubated in 300 μl of transport solution (HEPES buffer, pH 8, 10 μM 2‐deoxyglucose, 0.5 μCi/ml [^3^H]‐2‐deoxyglucose) for 5 min at 37°C and 2‐deoxyglucose transport calculated as described previously (Moghei et al., 2016; Somwar et al., 1998). Following the 5‐min incubation, plates were placed on ice and cells were washed with ice‐cold saline. They were then lysed with 1 ml of cold 0.05 M NaOH. An aliquot of the lysate was used to determine protein concentration while another aliquot was counted. Glucose transport was calculated by dividing the radioactivity counts by the specific activity of the transport solution to obtain pmol of deoxyglucose transported. This value was then divided by the protein concentration in each well to obtain pmol of deoxyglucose transported per μg protein.

### 2.5 Amino acid concentrations

Following treatments, myotubes were washed 2× in PBS, then harvested with 10% trichloroacetic acid. Lysates were centrifuged at 2.3 ***g*** for 15 min. Supernatant containing free amino acids were neutralized in a 1:2:1:8 ratio (sample: potassium phosphate buffer: 0.1 N hydrochloric acid: HPLC grade water, respectively). Neutralized samples were pre‐column derivatized with a 1:1 ratio of sample to o‐Phthalaldehyde (29.28 mM). They were then injected into a YMC‐Triart C18 column (C18, 1.9 μm, 75 × 3.0 mm; YMC America, Allentown, PA, USA) fitted onto an ultra‐high‐pressure liquid chromatography (UHPLC) system (Nexera X2, Shimadzu, Kyoto, Japan) that was connected to a fluorescence detector (Shimadzu, Kyoto, Japan; excitation: 340 nm; emission: 455 nm). Amino acids were eluted with a gradient solution derived from 20 mM potassium phosphate buffer (6.5 pH) mobile phase (mobile phase A) and a solution made from 45% acetonitrile, 40% methanol and 15% HPLC grade water (mobile phase B) at a flow rate of 0.8 ml/min. We used a gradient of 5%–100% of mobile phase B over 21 min. Amino acid concentrations were calculated using amino acid standard curves and were normalized to total protein.

### 2.6 Western blot analysis

Following treatments, cells were processed as described previously (Jeganathan et al., 2014; Zargar et al., 2011). Briefly, cells were harvested in lysis buffer (1 mM EDTA, 2% sodium dodecyl sulfate (SDS), 25 mM Tris‐HCl, pH 7.5, 1 mM DTT, and 10 μl/ml of each of protease inhibitor and phosphatase inhibitor cocktails). Proteins were separated on 10% SDS‐polyacrylamide gel electrophoresis (SDS‐PAGE) followed by transfer onto polyvinylidene difluoride (PVDF) membranes. Incubation in primary and secondary antibodies, image acquisition, and quantification were as described (Jeganathan et al., 2014; Zargar et al., 2011).

### 2.7 Data presentation and statistical analysis

Glucose transport data are presented as pmol of 2‐deoxyglucose per μg protein. Proteins for western blots were adjusted for loading using γ‐tubulin values. Statistical analyses were performed using GraphPad Prism 7 software. Data are presented as mean ± SEM. One‐way analysis of variance (ANOVA) was used and Tukey's post‐hoc tests were done to measure statistically significant differences among means. Significance was determined as *p* < 0.05.

## 3 RESULTS

### 3.1 Effect of inflammation and KIC on insulin‐stimulated glucose transport

**FIGURE 1:**
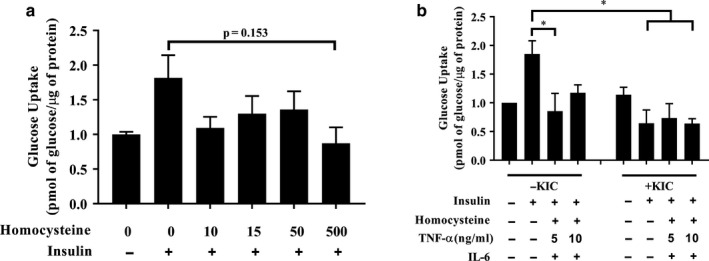
Effect of KIC and inflammatory factors on insulin‐stimulated glucose transport. L6 myoblasts were cultured in differentiation medium (DM) for 4 days. (a) On d 4 of differentiation, cells were incubated in DM containing varying concentrations of homocysteine (10–500 μM) for 24 hr. Incubation then continued in DM with homocysteine that was also supplemented with or without 100 nM insulin for 20 min, after which glucose transport assays were performed. (b) On d 4 of differentiation, cells were incubated with homocysteine (50 μM), IL‐6 (10 ng/ml), and TNF‐α (5 or 10 ng/ml) for 48 hr. Incubation then continued in the medium with pro‐inflammatory factors that was also supplemented without (−KIC) or with 200 μM KIC (+KIC) for 30 min. After, cells were incubated with or without 100 nM insulin for 20 min followed by glucose transport assay. Glucose transport is expressed as picomole of 2‐deoxyglucose transported into the cell/μg protein, and normalized to the no insulin (‐insulin) group. Data are mean ± SEM; n = 3–4 independent experiments (in which cells from different passages and/or different batches of cells were used in the independent experiments), with three technical replicates per experiment (number of wells per treatment condition per experiment); *p < 0.05 versus insulin group in the ‐KIC condition

Homocysteine at up to 500 μM had no effect on insulin‐stimulated glucose transport (Figure 1a), but a combination of inflammatory factors, including homocysteine, IL‐6, and TNF‐α (5 ng/ml) suppressed insulin‐stimulated glucose transport (*p* < 0.05, Figure 1b). KIC significantly reduced insulin‐stimulated glucose transport (*p* < 0.05, Figure 1b); co‐incubation with pro‐inflammatory factors did not have any further effect (Figure 1b).

### 3.2 BCAT2 depletion abolished the effect of KIC and/inflammatory factors on insulin‐stimulated glucose transport

**FIGURE 2:**
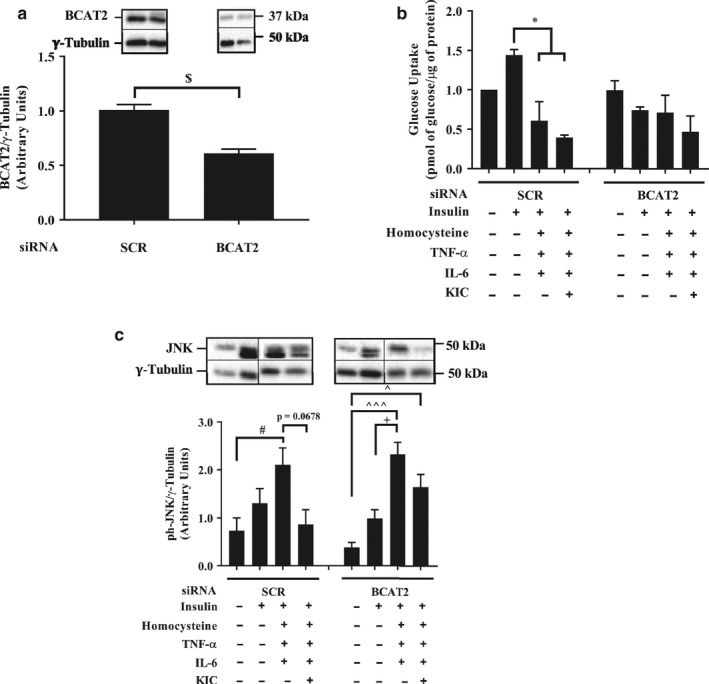
The effects of KIC and inflammatory factors on glucose transport are attenuated in BCAT2‐depleted cells. On d 3 of differentiation, cells were transfected with control (SCR) or BCAT2 siRNA oligonucleotides. Twenty‐four hours later, myotubes were incubated in DM that contained TNF‐α (10 ng/ml), IL‐6 (10 ng/ml) and homocysteine (50 μM) for 48 hr. Then, cells were treated with KIC and insulin as described in Figure 1. They were then harvested, or used for glucose transport assay as described in Figure 1. Proteins in lysates were immunoblotted against (a) BCAT2 and (c) phosphorylated (ph) SAPK/JNK^thr183/tyr185^. (b) Glucose transport, expressed as picomole of 2‐deoxyglucose transported into the cell/μg protein and normalized to the no insulin (‐insulin) in SCR condition. Mean ± SEM; n = 3 independent experiments with three technical replicates per experiment; $*p* < 0.05 versus SCR group; #*p* < 0.05, ###*p* < 0.001 versus no insulin in SCR condition; *p < 0.05 versus only insulin in SCR condition; ^*p* < 0.05, ^^^*p* < 0.001 versus no insulin in BCAT2 knocked‐down condition; +*p* < 0.05 versus insulin group in BCAT2 knocked‐down condition

**FIGURE 3:**
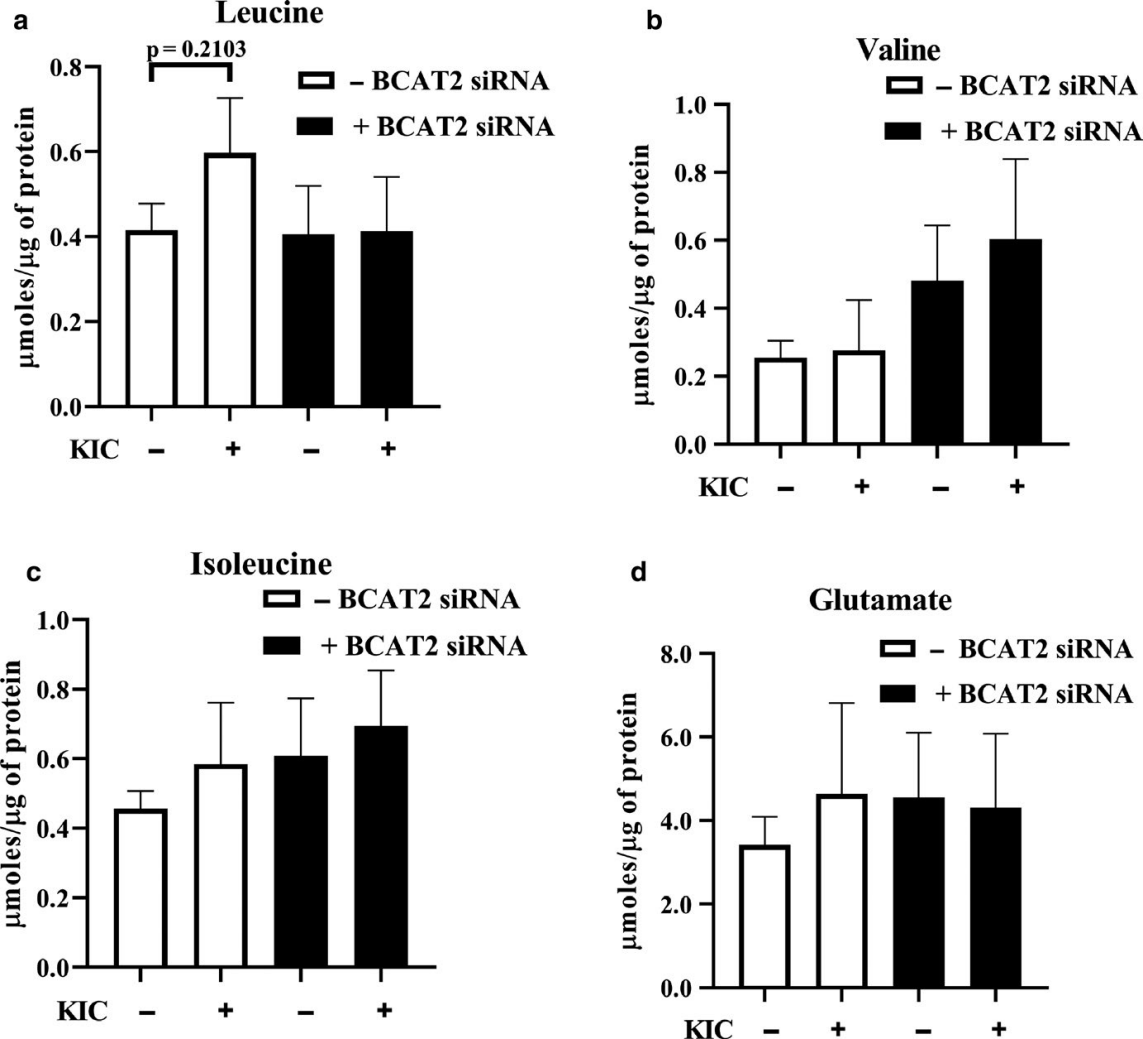
Effect of KIC supplementation and BCAT2 depletion on leucine, valine, isoleucine, and glutamate concentrations. On d 3 of differentiation, cells were transfected with control (SCR) siRNA or BCAT2 siRNA oligonucleotides. Forty‐eight hours post transfection, myotubes were starved of serum‐ and amino acid for 3 hr, then supplemented without (−KIC) or with 200 μM KIC (+KIC) for 1 hr. Cells were then harvested for amino acid analysis using HPLC. Amino acid concentrations are expressed as μmol of amino acid/μg protein. (a–d) Mean ± SEM; n = 3 independent experiments with three technical replicates per experiment

We previously showed that BCAT2 depletion abolished the effects of KIC on glucose transport (Moghei et al., 2016). We examined if this held true in an inflammatory environment. Consistent with our previous report, in BCAT2‐depleted myotubes (Figure 2b, Figure S1a), suppression of insulin‐stimulated glucose transport by the inflammatory factors was attenuated (Figure 2b). The presence of inflammation was confirmed with a significant increase in JNK phosphorylation (Figure 2c, Figure S1b), a marker of inflammation (Nieto‐Vazquez et al., 2008). This suggests that even in the context of inflammation, KIC was converted back into leucine to suppress insulin‐stimulated glucose transport. This is consistent with the observation that in myotubes with normal level of BCAT2, leucine intracellular concentrations, but not the concentration of valine, isoleucine, or glutamate tended to increase (~30%) in KIC‐treated cells (Figure 3a–d).

### 3.3 In inflammation, KIC does not affect glycogen synthase, Akt, S6K1, or S6 phosphorylation

**FIGURE 4:**
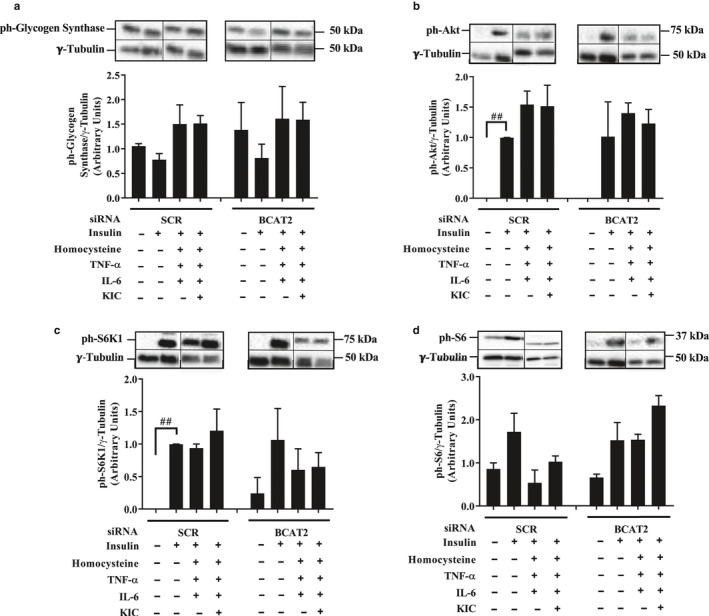
Effect of KIC, inflammation, and BCAT2 depletion on glycogen synthase, Akt, S6K1, and S6 phosphorylation. Cells were transfected with control (SCR) or BCAT2 siRNA oligonucleotides as described in Figure 2. Twenty‐four hour post transfection, myotubes were incubated in DM that contained TNF‐α (10 ng/ml), IL‐6 (10 ng/ml), and homocysteine (50 μM) for 48 hr. Cells were then treated with KIC and insulin as described in Figure 2. They were harvested and proteins immunoblotted against (a) ph‐Glycogen synthase^Ser641^, (b) ph‐Akt^Ser473^, (c) ph‐S6K1 ^Thr389^, and (d) ph‐S6^Ser235/236^. (a–d) Mean ± SEM; n = 3 independent experiments with three technical replicates per experiment; ##*p* < 0.01 versus no insulin in SCR condition

There were no significant treatment effects on glycogen synthase phosphorylation (Figure 4a, Figure S2a). We measured the effect of KIC and inflammatory factors on Akt phosphorylation, as Akt is important for the translocation of GLUT4, an insulin‐stimulated glucose transporter, from intracellular vesicles to the plasma membrane (Beg et al., 2017). Insulin significantly increased the phosphorylation (activation) of Akt in the SCR condition but there was no significant effect of KIC or inflammatory factors or BCAT2 depletion on this measure (Figure 4b, Figure S2b). Because S6K1 can catalyze the inhibitory serine phosphorylation of insulin receptor substrate‐1 (IRS‐1) and thereby induce insulin resistance (Gual et al., 2005; Moghei et al., 2016), we examined the phosphorylation of this kinase. Although insulin increased the phosphorylation of S6K1 (Figure 4c, Figure S2a) and tended to increase the phosphorylation of its substrate (ribosomal protein S6, Figure 4d, Figure S2b), neither KIC nor the inflammatory factors nor BCAT2 depletion affected these measures.

### 3.4 Effect of BCKD depletion and KIC supplementation on insulin‐stimulated glucose transport and insulin signaling

**FIGURE 5:**
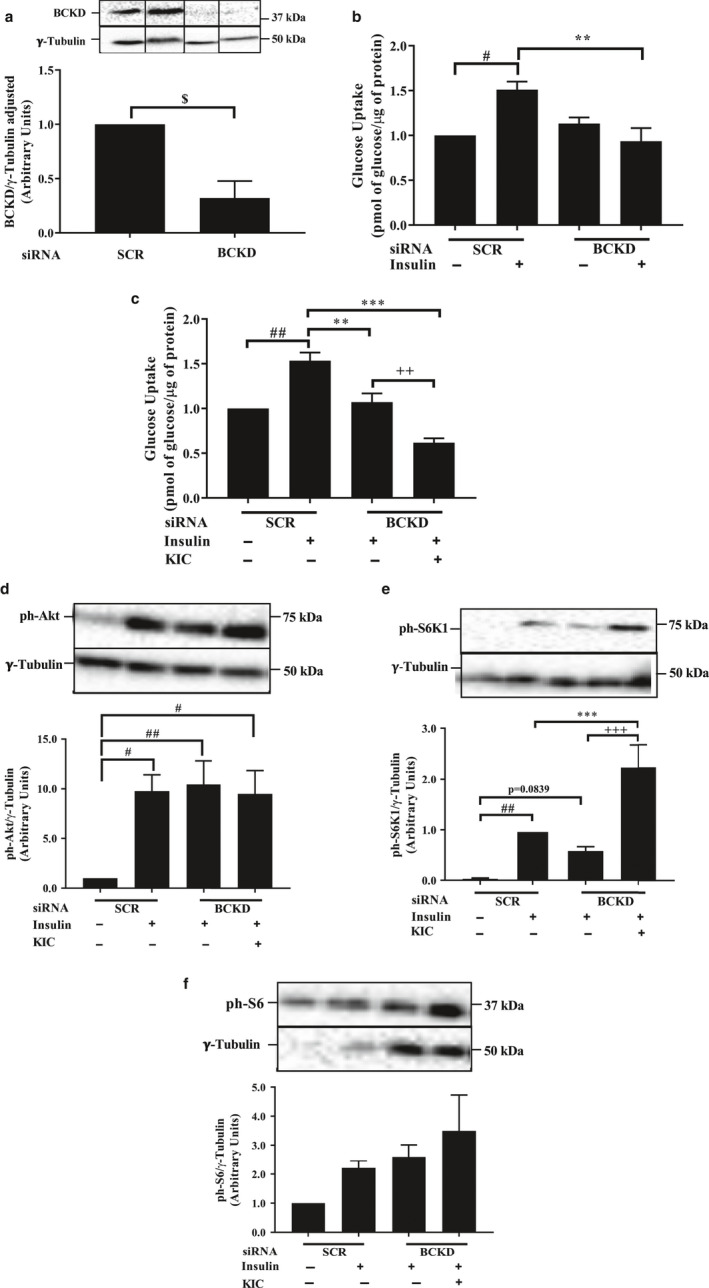
Effect of BCKD depletion on insulin‐stimulated glucose transport and insulin signaling. On d 4 of differentiation, cells were transfected with control (SCR) or BCKD siRNA oligonucleotides. Forty‐eight hours post transfection, myotubes were starved for 3 hr in serum‐ and amino acid‐free RPMI medium. Cells were then supplemented without (−KIC) or with 200 μM KIC (+KIC) for 30 min. After, cells were incubated with (+insulin) or without (−insulin) insulin (100 nM) for 20 min. They were then harvested or used for glucose transport assay. Proteins in lysates were immunoblotted against (a) BCKD, (d) ph‐Akt^ser473^, (e) ph‐S6K1 ^Thr389^, and (f) ph‐S6^ser235/236^. (b, c) Glucose transport, expressed as picomole of 2‐deoxyglucose transported into the cell/μg protein and normalized to the SCR, no insulin group (−insulin). Data for BCKD, ph‐Akt, and ph‐S6 were normalized to no insulin in SCR condition, while for S6K1 data were normalized to the insulin group in SCR condition due to a lack of signal in the no insulin in SCR condition. (a–f) Mean ± SEM; n = 4 independent experiments with three technical replicates per experiment; $*p* < 0.05, versus SCR; #*p* < 0.05, ##*p* < 0.01 versus no insulin in SCR condition; *p < 0.05, **p < 0.01, ***p < 0.001 versus insulin in SCR condition; ++*p* < 0.01, +++*p* < 0.001 versus insulin in BCKD condition

Whether reduced BCKD level causes insulin resistance has not been tested before. Depletion of BCKD‐E1α (Figure 5a, Figure S3a) suppressed insulin‐stimulated glucose transport by ~38% (*p* < 0.05, Figure 5b). Addition of KIC to the incubation medium further suppressed glucose transport (a ~ 42% decrease compared to the effect BCKD depletion alone, *p* < 0.05, Figure 5c). BCKD depletion and/or KIC supplementation did not have a significant effect on insulin‐stimulated phosphorylation of Akt (Figure 5d, Figure S3b). KIC increased insulin‐stimulated phosphorylation of S6K1, but not of S6, in BCKD‐depleted cells (*p* < 0.001, Figure 5e,f, Figure S3c).

## 4 DISCUSSION

While previous studies show a link between elevated levels of BCAA and their metabolites on insulin resistance, an important question has been to ascertain whether increased circulating levels of BCAA/BCAA metabolites cause insulin resistance, or merely reflect insulin resistance. To answer this question, it is critical to study the effects of these amino acids and their metabolites in conditions that mimic insulin resistance. Here, we have demonstrated that (a) incubation of myotubes in KIC, or in a combination inflammatory factors suppressed insulin‐stimulated glucose uptake; (b) addition of the inflammatory factors did not worsen the effect of KIC; (c) the effect of the inflammatory factors, alone or combined with KIC, on insulin‐stimulated glucose transport was abrogated in myotubes depleted of BCAT2; (d) myotubes depleted of a critical subunit of BCKD had impaired insulin‐stimulated glucose transport, especially when the cells were also incubated in KIC. Collectively, these data point to a critical role for muscle BCAA catabolism in the regulation of muscle glucose transport and, by implication, regulation of whole‐body glucose metabolism.

Many correlational analysis (Felig et al., 1969; Giesbertz et al., 2015; Mccormack et al., 2013; Perng et al., 2014; Wang et al., 2011) and mechanistic (Lackey et al., 2013; Newgard, 2012; Newgard et al., 2009) studies show a positive relationship between increased levels of circulating BCAA and BCAA metabolite, and insulin resistance. We and others have shown that BCAA (especially leucine) (Baum et al., 2005; Moghei et al., 2016; Tremblay & Marette, 2001) and metabolites of BCAA (Jang et al., 2016; Moghei et al., 2016) can induce insulin resistance of glucose transport in skeletal muscle/muscle cells. These pieces of evidence suggest that irrespective of the reasons behind the elevation in circulating BCAA/BCAA metabolites, these compounds can in themselves cause or worsen insulin resistance in skeletal muscle.

An equally important issue is the reason for the elevation in BCAA/BCAA metabolites in insulin resistance. Increase in circulating BCAA may arise due to increased consumption and/or intestinal absorption and release of the amino acids into systemic circulation. Reduced BCAA consumption improves insulin sensitivity (Cummings et al., 2018; White et al., 2016). The increase in circulating BCAA/BCAA metabolites may also be due to impaired utilization, for example, due to reduced protein synthesis and/or increased proteolysis. Consistent with this, skeletal muscle (ViM & Garlick, 1975) and whole‐body protein synthesis (Pereira et al., 2008) are reduced in insulin resistance and in type 2 diabetic individuals. Another possibility is impaired catabolism of these amino acids. In line with this, mRNA levels of the E1β subunit of BCKD are reduced in skeletal muscle of T2DM patients (Hernández‐Alvarez et al., 2017). Also, BCKD activity is downregulated in skeletal muscle, liver and adipose tissue of type 2 diabetic mice (Lian et al., 2015). Interestingly, although mice lacking BCAT2 have elevated BCAA levels, they did not manifest insulin resistance (She et al., 2007). Inhibition of BCKD kinase (BDK), the enzyme that phosphorylates and inhibits BCKD, leads to reduced levels of BCAA and improved whole‐body insulin sensitivity (White et al., 2018; Zhou et al., 2019). Here, we demonstrated that depletion of E1α subunit of BCKD impaired insulin‐stimulated glucose uptake, which worsened in the presence of KIC. Together, these findings indicate that not only can metabolites of BCAA cause insulin resistance, reduced levels/activity of the enzymes involved in the catabolism of these amino acids too may be causative.

Insulin resistance in skeletal muscle is a main causative abnormality in the development of type 2 diabetes (DeFronzo & Tripathy, 2009). Although BCAT2 activity is high in muscle (Brosnan & Brosnan, 2006; Suryawan et al., 1998), it is generally thought that catabolism of BCAA beyond the formation of BCKA occurs predominantly in liver and adipose tissue (Harper et al., 1984; Herman et al., 2010), but with little activity in skeletal muscle (Harper et al., 1984). Nevertheless, increased muscle intracellular levels of KIC, isovaleric CoA, and of other BCAA metabolites are seen in insulin resistance (Giesbertz et al., 2015; Lynch & Adams, 2014; Newgard et al., 2009; Perng et al., 2014). In addition, mice with muscle‐specific deletion of BDK have 60% and 40% reductions in muscle BCAA and plasma BCAA levels, respectively (Ishikawa et al., 2016). Furthermore, 3‐hydroxyisobutyrate (3‐HIB), a catabolic intermediate of the BCAA valine that is released from muscle, promotes insulin resistance by stimulating muscle uptake and accumulation of fatty acids (Jang et al., 2016). Our data showing that depletion of E1α subunit of BCKD in myotubes impaired insulin‐stimulated glucose transport are consistent with these data and suggest that impaired catabolism of BCAA in skeletal muscle, which would lead to the accumulation of BCAA intermediates, can cause insulin resistance.

Controversy still exists about the role of leucine in the pathogenesis of insulin resistance. Leucine is associated with insulin resistance and a higher risk of T2DM (Adams, 2011; Lynch & Adams, 2014; Mccormack et al., 2013; Newgard et al., 2009), however, studies have also shown improved insulin sensitivity with leucine supplementation in vivo (Binder et al., 2013; Macotela et al., 2011; Nishitani et al., 2002 *)*. Metabolomics studies have also linked elevated KIC levels to insulin resistance and T2DM (Adams, 2011; Mccormack et al., 2013; Newgard et al., 2009). High levels of KIC is associated with insulin resistance in humans (Perng et al., 2014) and animals (Giesbertz et al., 2015). On the other hand, leucine and KIC at supraphysiological concentrations stimulate glucose uptake in soleus muscle, but the effect of leucine is greater (Nishitani et al., 2002). Also, supplementation with leucine or KIC stimulates insulin secretion in vivo, but the conversion of KIC to leucine is required for the KIC effect (Zhou et al., 2010). Much less is known about the effect of KIC in the pathogenesis of insulin resistance *in vivo* but our work (this study and [Moghei et al., 2016]) and the studies cited above (Adams, 2011; Lynch & Adams, 2014; Mccormack et al., 2013; Newgard et al., 2009) would suggest a greater role for leucine compared to KIC in the pathogenesis of insulin resistance.

There are elevated levels of circulating inflammatory factors in insulin resistance (Singh & Saxena, 2010). It is possible therefore that the effects of BCAA and their metabolites on insulin‐stimulated glucose uptake might be affected by these factors. At the concentrations tested, homocysteine by itself did not affect glucose transport. This might be because, unlike in adipocytes, muscle is not a source of resistin, an adipocyte hormone that may mediate or promote the effect of homocysteine in causing adipocyte insulin resistance (Li et al., 2008). Although incubation of myotubes in a mix containing homocysteine, IL6, and TNF‐α suppressed glucose uptake, no further suppression was observed when KIC was also added. It is possible that suppression of glucose transport was already maximal with either KIC or the inflammatory factors used. It would be interesting to study whether inflammatory factors alter BCAA catabolism and if such alterations mediate the effects of these factors on insulin sensitivity.

Previous reports point to mammalian/mechanistic target of rapamycin complex 1 (mTORC1) as mediating the effect of BCAA and their metabolites on insulin resistance (Newgard, 2012; Newgard et al., 2009; Um et al., 2004, 2006). This is because activated mTORC1/S6K1 phosphorylates serine residues of IRS‐1 (S302, S307, S612, and S1101) (Gual et al., 2005). This leads to downregulation of IRS‐1 (Pederson et al., 2001) and/or inhibits IRS‐1 signaling (Lynch & Adams, 2014; Yoon, 2016). Similar to these reports, we observed a trend toward increased S6K1 phosphorylation in cells incubated with both KIC and the pro‐inflammatory factors, an effect that was attenuated when BCAT2 was knocked down, consistent with our previous work (Moghei et al., 2016). Interestingly, compared to myotubes that were not treated with KIC, S6K1 phosphorylation was substantially higher in BCKD‐E1α‐depleted cells that were treated with KIC. This might be a result of KIC and possibly leucine accumulation in those cells.

A limitation of this work is that it is an in vitro study. It is necessary to confirm whether observation made here regarding the effect of KIC, with or without treatment with inflammatory factors, and BCKD depletion can be reproduced in intact animals. Nevertheless, our study examining the direct effect of KIC and inflammation, and BCKD depletion in myotubes complements such *in vivo* studies, as examining direct effects of substrates on a single tissue in *vivo* would be challenging due to the contributions of other tissues, growth factors, and metabolites.

In conclusion, we demonstrated that incubation of myotubes with inflammatory factors, with or without KIC, suppressed insulin‐stimulated glucose uptake, but this effect was attenuated when cells were depleted of BCAT2. Cells depleted of E1α subunit of BCKD had reduced insulin‐stimulated glucose uptake, especially in combination with KIC treatment. Collectively, our data suggest that reduced levels/activity of enzymes in BCAA catabolic pathway have a causative effect on insulin resistance, and therefore regulation of these enzymes may have a therapeutic potential for the management of insulin resistance. These data also suggest that interventions that improve insulin sensitivity, such as endurance exercise (Lee et al., 2018; Wagenmakers et al., 1989), likely do so via increased BCKD level/BCAA catabolism (Shimomura et al., 2004; Shimomuras et al., 2006; Wagenmakers et al., 1989).

## CONFLICT OF INTEREST

The authors declare no conflict of interest.

## AUTHOR CONTRIBUTIONS

GM and OAJA conceived and designed the experiments. GM performed the experiments and drafted the initial version of the manuscript. OAJA reviewed and edited the manuscript. Both authors approved the final version of the manuscript.

## Supporting information



Supplementary Material
