# Supplementary material for: Effects of ketoisocaproic acid and inflammation on glucose transport in muscle cells
Source: Physiol Rep. 2021 Jan 5;9(1):e14673. doi: 10.14814/phy2.14673 (PMC7785050; doi:10.14814/phy2.14673)

Supp Fig 1A, related to Fig 2a:

On d 3 of differentiation, cells were transfected with control (SCR) or BCAT2 siRNA oligonucleotides. Twenty-four h later, myotubes were incubated for 48 h in DM that contained TNF-α (10 ng/ml), IL-6 (10 ng/ml), and homocysteine (50 μM). Then, cells were treated with KIC and insulin as described in Fig 1. They were then harvested and proteins in lysates immunoblotted against the indicated antibodies (Supp Fig 1A, 1B).

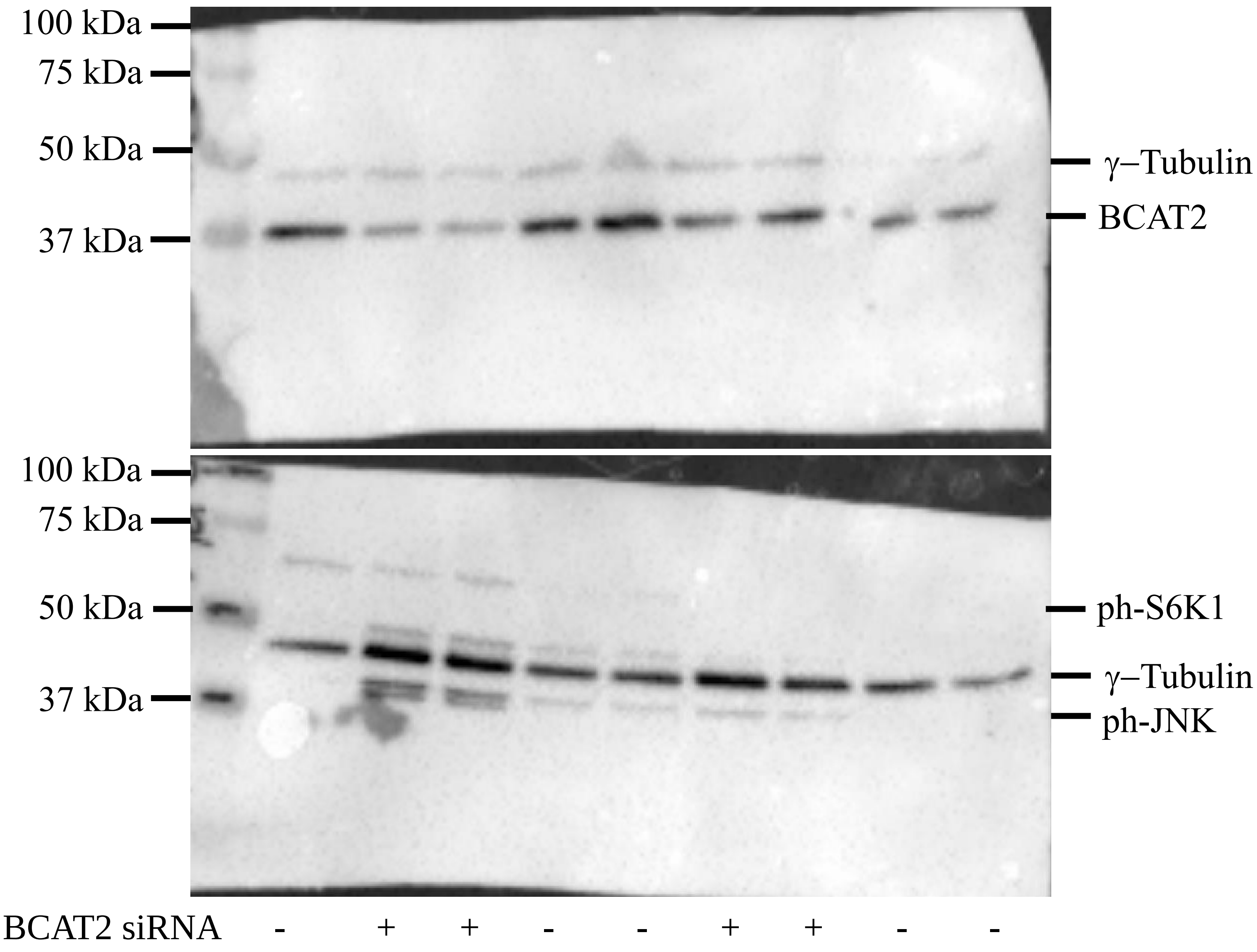

Supp Fig 1B, related to Fig 2C

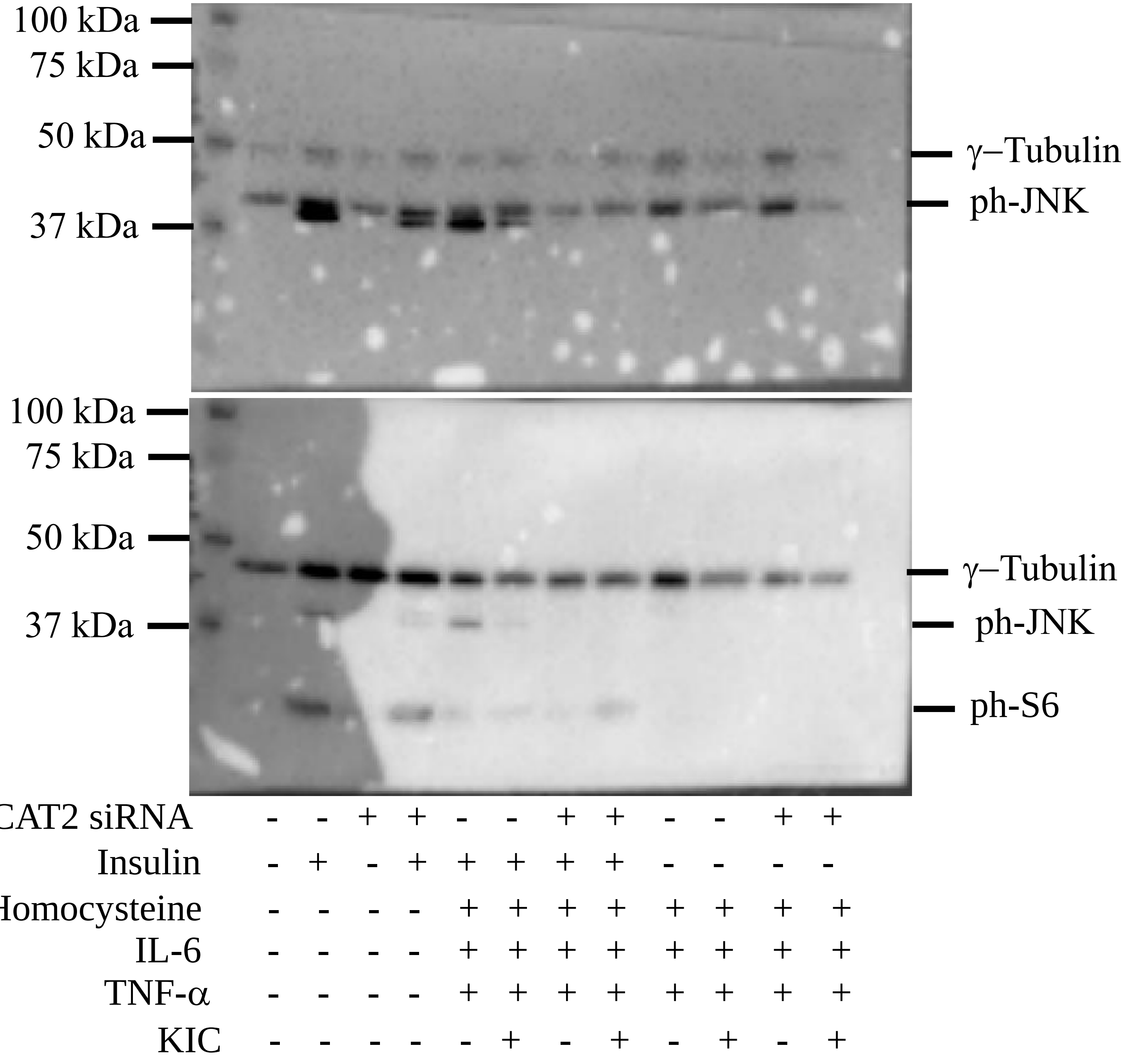

Supp Fig 2A, related to Fig 4A, 4C:
Cells were transfected with control (SCR) or BCAT2 siRNA oligonucleotides as described in Fig 2. Twenty-four h post transfection, myotubes were incubated for 48 h in DM that contained the inflammatory factors. Cells were then treated with KIC and insulin as described in Fig 2. They were harvested and proteins immunoblotted against the indicated antibodies (Supp Fig 2A, 2B).

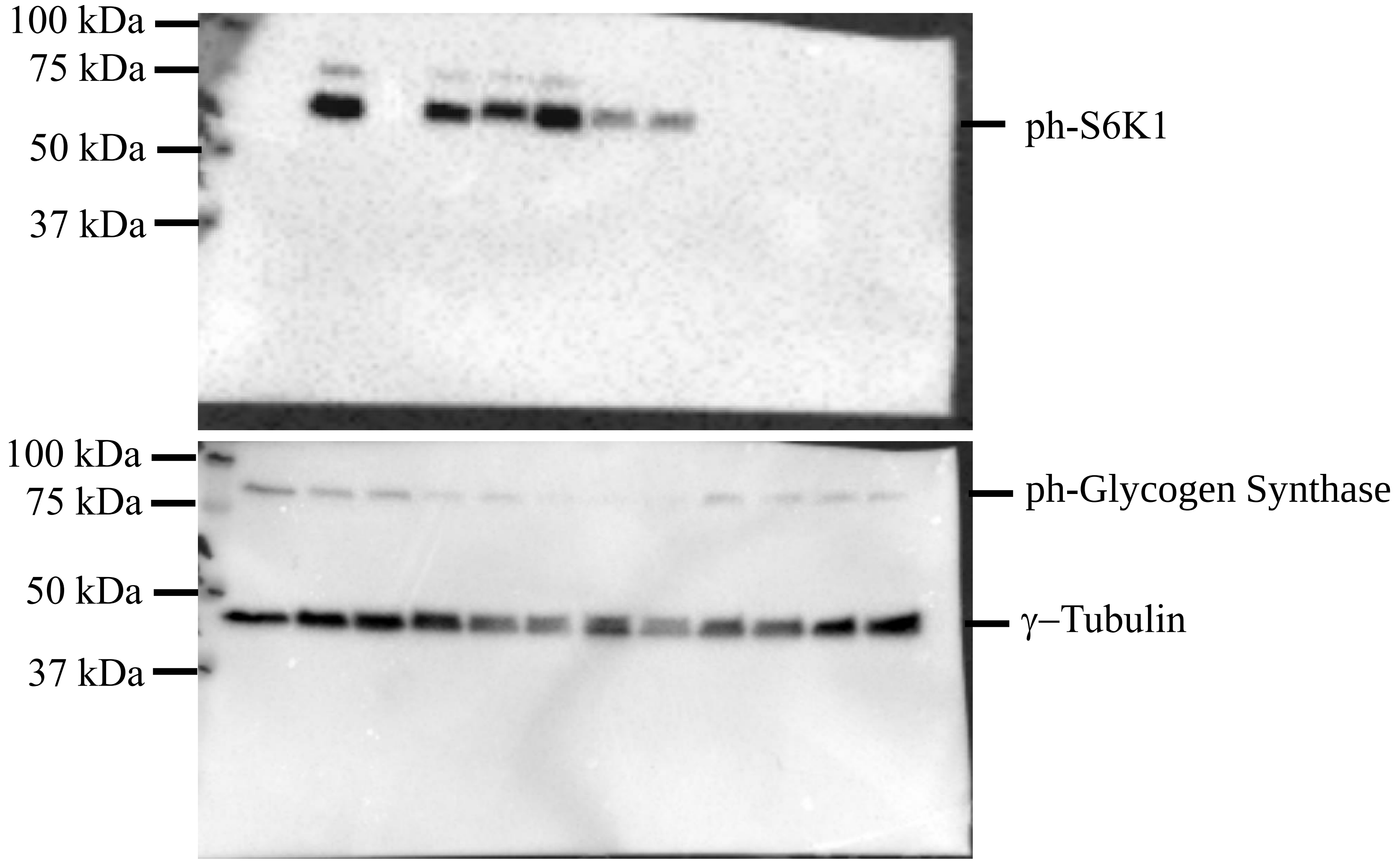

BCAT2 siRNA	-	-	+	+	-	-	+	+	-	-	+	+
Insulin	-	+	-	+	+	+	+	+	-	-	-	-
Homocysteine	-	-	-	-	+	+	+	+	+	+	+	+
IL-6	-	-	-	-	+	+	+	+	+	+	+	+
TNF-α	-	-	-	-	+	+	+	+	+	+	+	+
KIC	-	-	-	-	-	+	-	+	-	+	-	+

Supp Fig 2B, related to Fig 4B and 4D

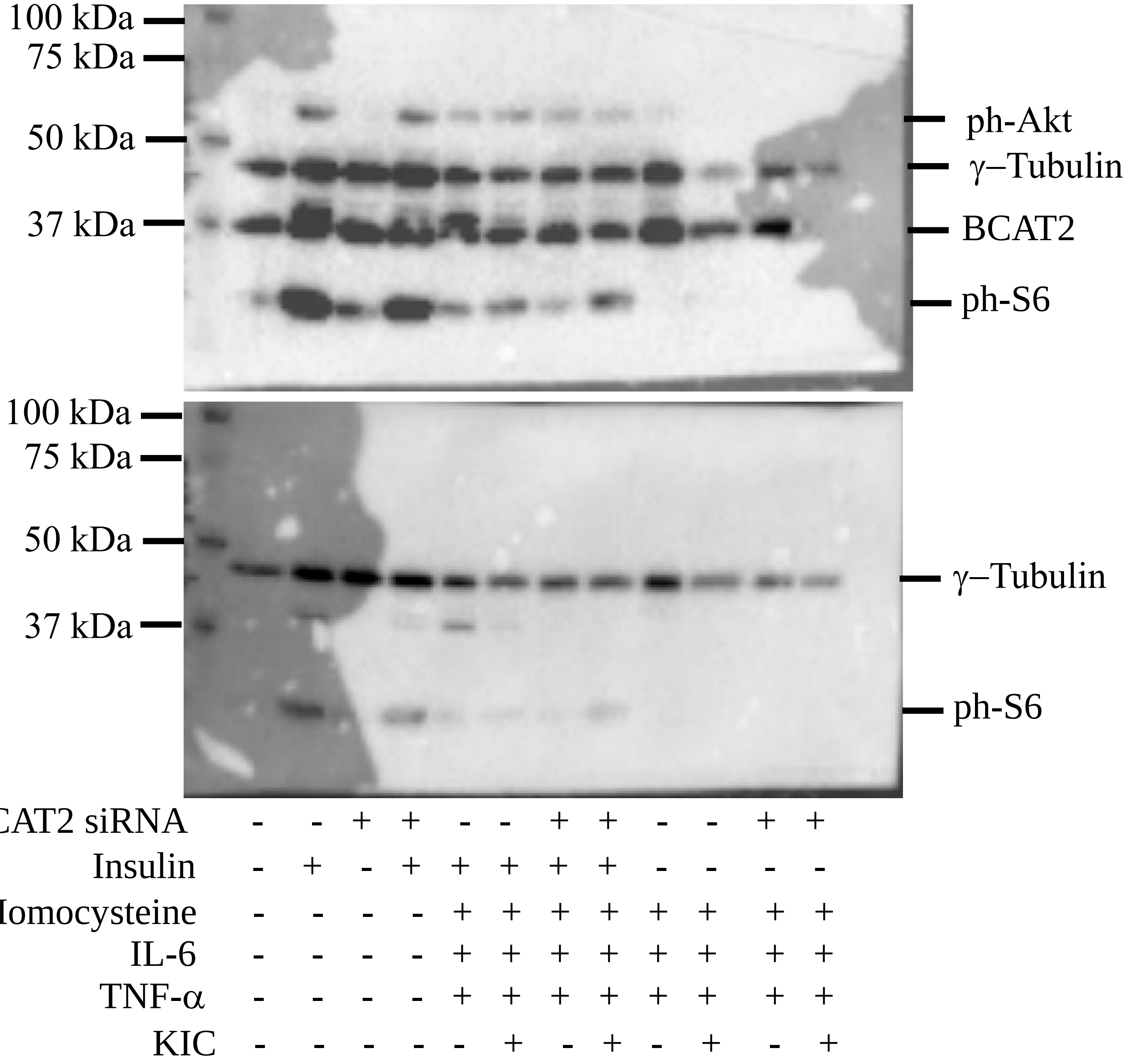

Supp Fig 3A, related to Fig 5A:

On d 4 of differentiation, cells were transfected with control (SCR) or BCKD siRNA oligonucleotides. Forty-eight h post transfection, myotubes were starved for 3 h in serum- and amino acid-free RPMI medium. Cells were then supplemented with or without KIC for 30 min. After, cells were incubated with or without insulin for 20 min. Proteins in lysates were immunoblotted against the indicated antibodies (Supp Fig 3A-3C).

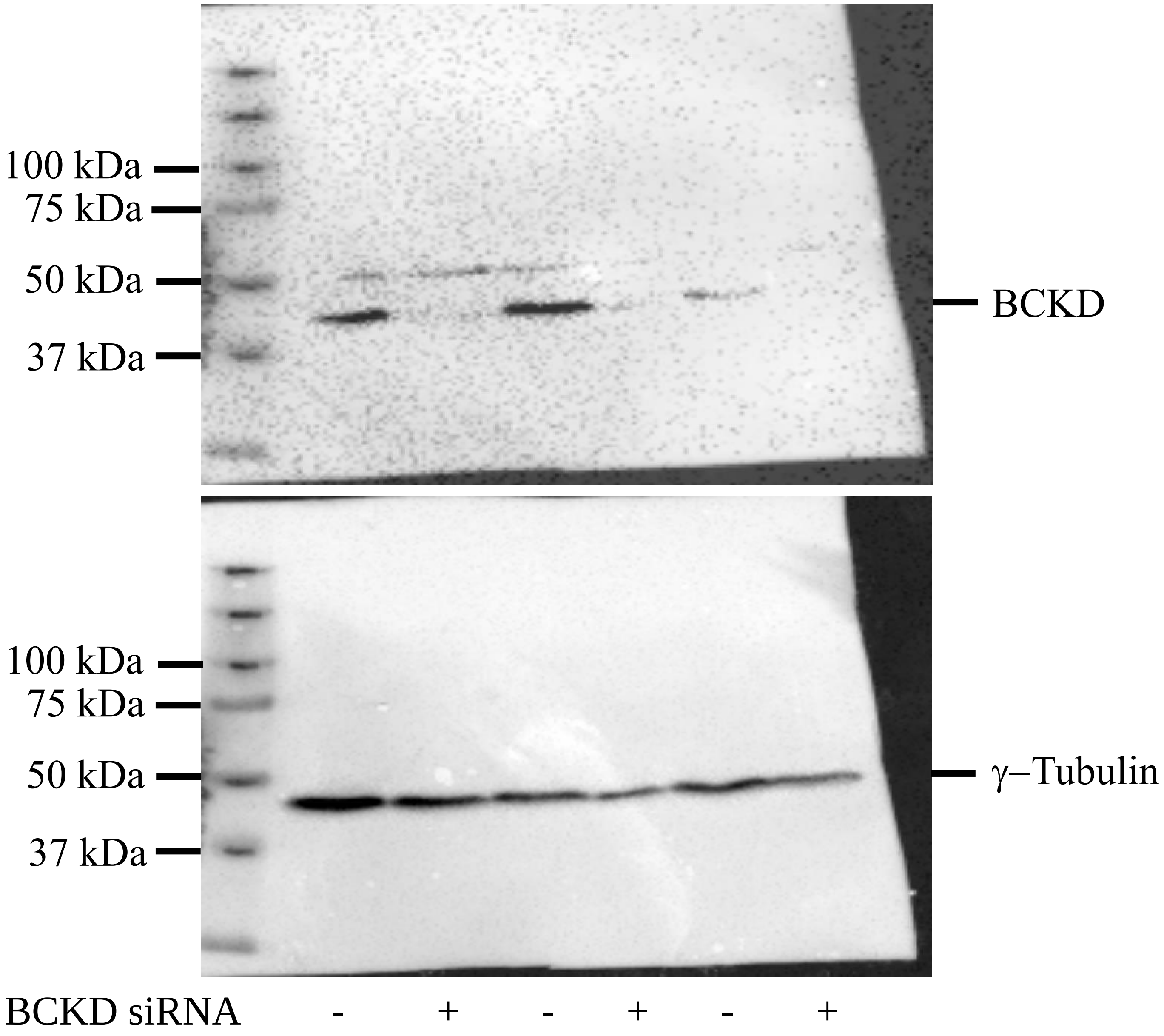

Supp Fig 3B, related to Fig 5D

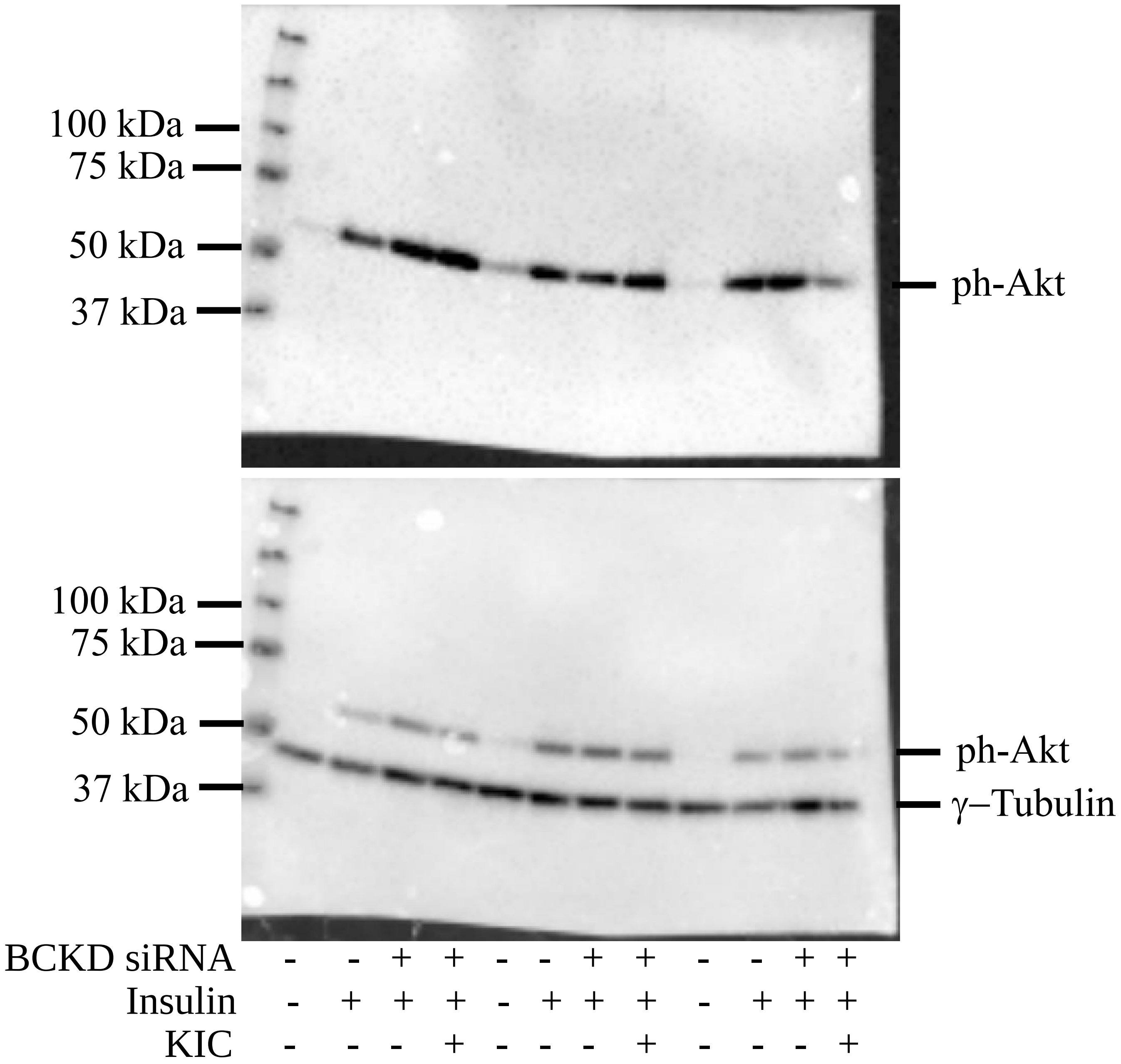

Supp Fig 3C, related to Fig 5E, 5F

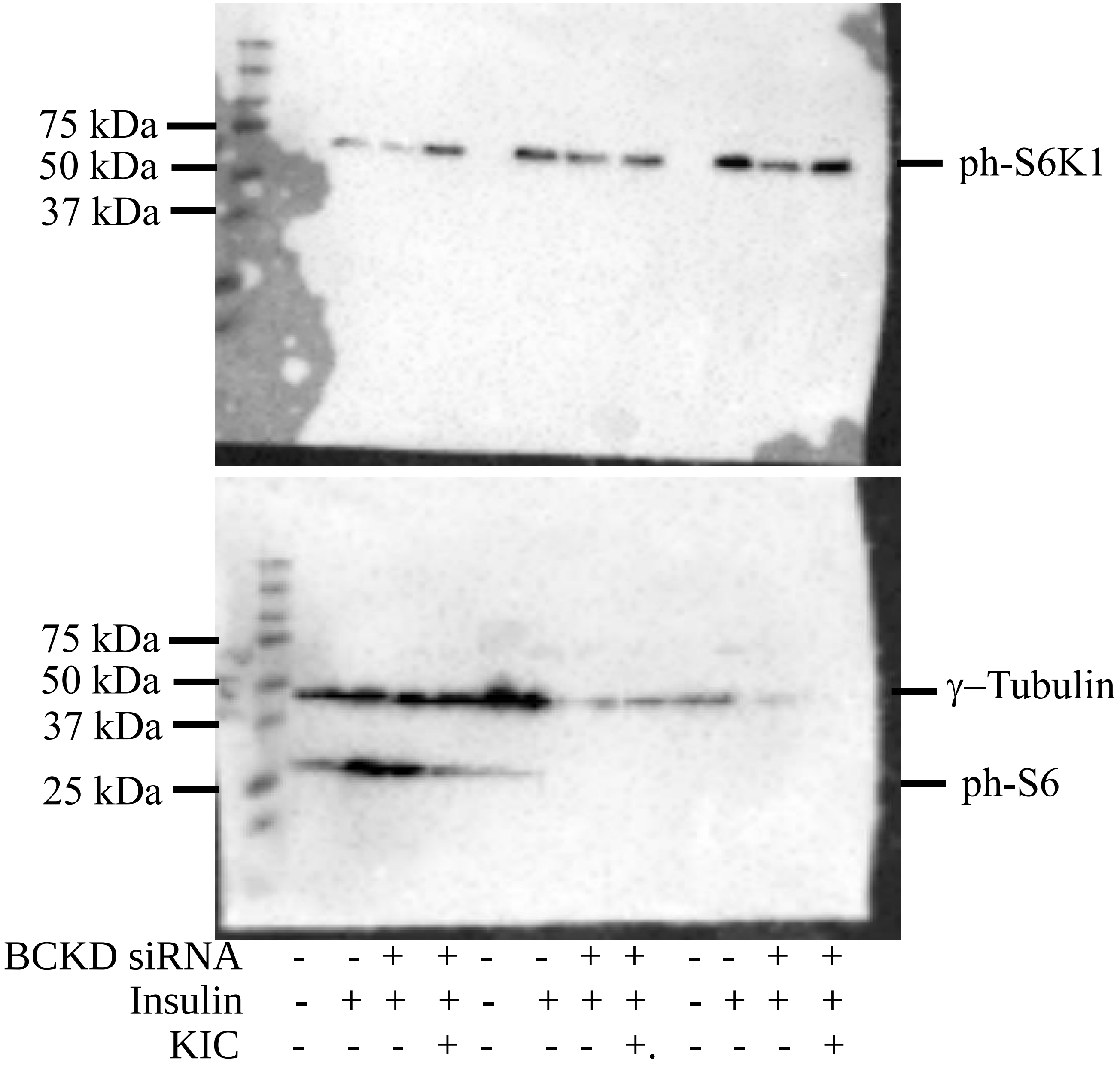

Supplement: Supplementary file 1 — Supplementary Material [file PHY2-9-e14673-s001.pdf]
